# Association between antithrombin levels and prognosis in patients with sepsis: a retrospective cohort study based on the MIMIC-IV and MIMIC-III databases

**DOI:** 10.1186/s40560-026-00862-x

**Published:** 2026-02-02

**Authors:** Bingkui Ren, Gang Zhou, Haiyan Xue, Siying Chen, Yuping Zhang, Guangjie Wang, Fengxue Zhu

**Affiliations:** 1 Department of Intensive Care Medicine, Peking University People’s Hospital, No. 11, Xizhimen South Street, Xicheng District, Beijing, 100044 People’s Republic of China; 2 Chinese Academy of Medical Sciences and Peking Union Medical College, Beijing, 100730 People’s Republic of China

**Keywords:** Antithrombin, Sepsis, Mortality, Disseminated intravascular coagulation, Acute kidney disease

## Abstract

**Background:**

Sepsis is a critical determinant of mortality in critical patients. Antithrombin (AT) plays a pivotal role as a serine protease inhibitor with dual anticoagulant and anti-inflammatory functions, yet its precise role in prognostic stratification remains undefined. This study aimed to investigate the association between AT activity and clinical outcomes in sepsis and to identify critical prognostic thresholds.

**Methods:**

We conducted a retrospective cohort study of 222 septic patients from the MIMIC-IV and MIMIC-III databases. AT activity was measured within the first 24 h following sepsis diagnosis, with the primary outcome defined as 28-day all-cause mortality. For preliminary description, AT activity was categorized into tertiles. The primary analysis utilized restricted cubic splines (RCS) to model the dose–response relationship and identify risk thresholds. Multivariable Cox regression models were employed to adjust for demographics, comorbidities, and SOFA score. Subgroup and survival analyses were performed to evaluate effect modification and visualize outcome differences across threshold-defined risk groups. To visually compare survival outcomes between patient groups defined by the RCS-derived risk thresholds, we generated Kaplan–Meier curves and employed log-rank tests.

**Results:**

A non-linear relationship between AT activity and 28-day mortality was identified, with a marked increase in risk observed below approximately 55% in the overall cohort. Patients with AT activity < 55% had significantly higher 28-day mortality (34.2% vs. 14.4%, p = 0.001), ICU mortality (33.3% vs. 9.0%, p < 0.001), and incidences of disseminated intravascular coagulation (DIC) (22.5% vs. 3.6%, p < 0.001) and acute kidney injury (AKI) (78.4% vs. 62.2%, p = 0.013). Subgroup analysis revealed a significant interaction with hypertension. In the hypertensive subgroup, a similarly elevated risk zone was observed below approximately 64% AT activity. Hypertensive patients below this level had markedly increased 28-day mortality (42.3% vs. 9.62%, p < 0.001), ICU mortality (38.5% vs. 5.77%, p < 0.001), and incidences of DIC (19.2% vs. 1.92%, p < 0.001).

**Conclusion:**

Reduced AT activity was significantly associated with higher mortality and organ dysfunction in sepsis. Risk thresholds were observed at approximately 55% for the overall cohort and 64% among hypertensive patients. Patients below these levels exhibited significantly increased mortality and higher incidences of DIC and AKI. These findings support AT activity as a prognostic biomarker for risk stratification and highlight its potential to inform future management strategies for high-risk patients.

**Supplementary Information:**

The online version contains supplementary material available at 10.1186/s40560-026-00862-x.

## Introduction

Sepsis is defined as a life-threatening organ dysfunction resulting from a dysregulated host response to infection [1]. It remains a major global health burden, contributing significantly to morbidity and mortality among critically ill patients and presenting considerable challenges for both clinical management and scientific investigation [2, 3]. In sepsis, dysregulation of the host immune response leads to concurrent activation of inflammatory and coagulation pathways, a condition clinically designated as sepsis-induced coagulopathy (SIC) [4]. The process begins when pathogen-associated molecular patterns induce tissue factor expression on immune cells, activating the coagulation cascade through FVIIa, FXa, thrombin, and fibrin [5]. These mediators promote microvascular thrombosis while amplifying inflammatory signaling via protease-activated receptors, creating a self-sustaining cycle [6]. In clinical practice, SIC frequently progresses to disseminated intravascular coagulation (DIC), resulting in multiple organ dysfunction or hemorrhage [7]. The limited success of targeted anticoagulant therapies in trials underscores the need for integrated strategies that simultaneously address the underlying infection and coagulation dysregulation [8].

Antithrombin (AT) is a hepatically synthesized, 58-kDa plasma glycoprotein, which acts as a serine protease inhibitor affecting multiple components of the intrinsic, extrinsic, and common coagulation pathways [9, 10]. AT levels are significantly altered during sepsis and are influenced by both the systemic inflammatory response and standard therapeutic interventions in critical care [11–13]. AT deficiency in sepsis is increasingly recognized as a key driver that elevates thrombotic risk and can propel the progression toward DIC and multi-organ failure [14–16].

The well-established role of AT deficiency in the pathogenesis of sepsis-induced coagulopathy has not yet translated into effective clinical therapies, as evidenced by inconsistent outcomes in supplementation trials. The KyberSept trial did not show a reduction in mortality from high-dose AT therapy in broad sepsis cohorts. Moreover, later meta-analyses reaffirmed this conclusion, indicating no sufficient evidence to recommend AT for routine care in critically ill patients with severe sepsis or septic shock [17, 18]. However, patients with established DIC constitute a specific high-risk subgroup in whom analyses suggest potential survival benefits, highlighting the inadequacy of uniform treatment strategies [19].

In this study, we aimed to investigate the association between AT activity and clinical outcomes in patients with sepsis, with a focus on identifying critical prognostic thresholds for mortality and organ dysfunction. Furthermore, we sought to evaluate the potential utility of AT as a stratification biomarker to guide future targeted management strategies, particularly in high-risk subgroups.

## Methods

### Data source

This retrospective cohort study utilized data from two critical care databases: the Medical Information Mart for Intensive Care IV (MIMIC-IV, version 3.1) and the MIMIC-III Clinical Database (CareVue subset, version 1.4). The MIMIC-III database contains comprehensive data from patients admitted to the intensive care units (ICUs) of the Beth Israel Deaconess Medical Center (BIDMC) between 2001 and 2012 [20]. The MIMIC-IV database, an updated and expanded version, includes over 70,000 ICU admissions at the same institution between 2008 and 2019 [21]. A significant challenge in combining these databases is the potential for overlapping patient records during the concurrent data collection period (2008–2012). Because subject identifiers were reassigned between MIMIC-III and MIMIC-IV, direct identification and removal of duplicate patients is not feasible. To address this, we employed the officially provided MIMIC-III CareVue subset. This subset intentionally excludes all patient data recorded after 2008, when the hospital transitioned to the MetaVision clinical information system, thereby ensuring a cohort of unique patients without overlap with those in MIMIC-IV [22]. The merger of these two complementary datasets creates a combined study cohort with a temporal range from 2001 to 2019, which not only increases the sample size but also enhances the generalizability of our findings across a broader timeframe.

The study received approval from the Institutional Review Boards (IRB) of the Massachusetts Institute of Technology (MIT) and the BIDMC, with a waiver of informed consent due to the retrospective nature of the research. The first author has completed the required CITI Program course, “CITI Data or Specimens Only Research” (Certification No. 48763426).

### Population selection

**Fig. 1:**
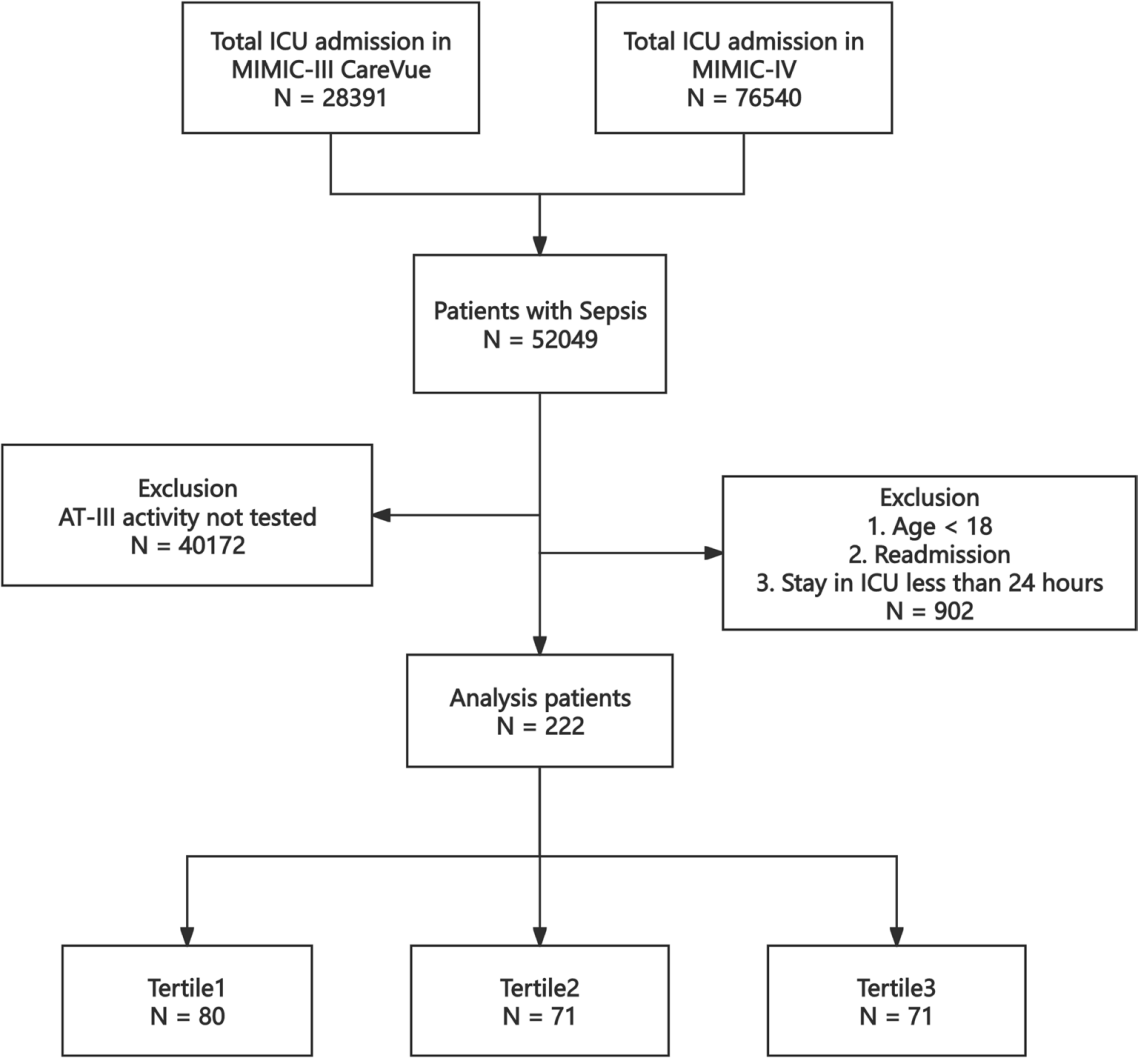
Study cohort flowchart

Patients were enrolled if they met the following criteria: (1) diagnosed with sepsis according to Sepsis 3.0 definition [23]; (2) patients in whom AT activity was assessed within the first 24 h after the diagnosis of sepsis; (3) aged ≥ 18 years; (4) stayed in the ICU for more than 24 h; (5) first stay was reserved if repetitively admitted into the ICU. Finally, a total of 222 patients were enrolled in this study and grouped into three groups based on the tertiles of the AT-III activity; please refer to Fig. 1 for the specific research process.

### Data extraction

Data extraction was performed using Structured Query Language (SQL) to retrieve clinical and laboratory parameters recorded within the first 24 h following a diagnosis of sepsis. The extracted data comprised patient demographics (sex, race, age); anthropometrics (weight, height, BMI); severity-of-illness scores, including the Acute Physiology Score III (APSIII), Simplified Acute Physiology Score II (SAPSII), Oxford Acute Severity of Illness Score (OASIS), Logistic Organ Dysfunction System (LODS), Sequential Organ Failure Assessment (SOFA) score, and Charlson Comorbidity Index (CCI); vital signs (heart rate, temperature, respiratory rate, mean arterial pressure, systolic and diastolic blood pressure); blood gas parameters (SpO₂, pH, PaO₂, bicarbonate); and laboratory values, which encompassed complete blood count (white blood cells, red blood cells, hemoglobin, platelets), metabolic panel (lactate, creatinine, blood urea nitrogen, glucose, calcium, chloride, sodium, potassium), liver function tests (alanine aminotransferase, aspartate aminotransferase, albumin, total bilirubin), and coagulation profile (International Normalized Ratio, prothrombin time, activated partial thromboplastin time). We also extracted data on comorbidities (heart failure, hypertension, atrial fibrillation, diabetes, chronic kidney disease, coronary artery disease, liver cirrhosis), and outcome measures, including ICU mortality, 28-day mortality, and the incidence of acute kidney injury (AKI) and DIC. The diagnosis of AKI was defined according to the Kidney Disease: Improving Global Outcomes (KDIGO) 2012 criteria [24], while DIC was diagnosed based on the International Society on Thrombosis and Haemostasis (ISTH) 2001 guidelines [25]. All these data stated above were extracted and can be seen in Table 1. We considered 28-day mortality to be the primary outcome. ICU mortality, DIC, and AKI were assessed as exploratory secondary outcomes. All laboratory variables were obtained only within the first 24 h after the diagnosis of sepsis.

**Table 1:** Characteristics and outcomes of participants categorized by AT activity

Characteristic	Overall	T1(< 46%)	T2(46–73%)	T3(> 73%)	p-value
	*N* = 222	*N* = 80	*N* = 71	*N* = 71	
Antithrombin (%)	54.5 [40.0;78.8]	34.0 [23.0;41.2]	55.0 [50.5;65.0]	89.0 [80.0;97.5]	< 0.001
Age (years)	53.5 [39.2;64.0]	54.0 [41.8;67.2]	53.0 [40.5;64.0]	50.0 [36.5;64.0]	0.315
Sex:					0.179
Female	81 (36.5%)	27 (33.8%)	22 (31.0%)	32 (45.1%)	
Male	141 (63.5%)	53 (66.2%)	49 (69.0%)	39 (54.9%)	
Race:					0.025
White	111 (50.0%)	50 (62.5%)	25 (35.2%)	36 (50.7%)	
Black	27 (12.2%)	5 (6.25%)	12 (16.9%)	10 (14.1%)	
Other	88 (39.6%)	25 (31.3%)	34(47.8%)	25 (35.2%)	
Weight (kg)	89.6 (22.4)	89.5 (20.9)	89.4 (22.6)	89.8 (24.0)	0.995
Height (cm)	175 [165;178]	173 [165;178]	175 [168;180]	173 [165;178]	0.871
BMI (kg/m^2^)	29.3 [25.2;34.0]	29.6 [25.0;33.6]	29.1 [25.7;34.2]	29.4 [25.2;34.1]	0.901
APSIII	57.5 [42.0;75.8]	70.5 [60.8;89.2]	54.0 [43.0;67.0]	45.0 [33.5;59.5]	< 0.001
SAPSII	40.0 [28.0;52.8]	48.0 [38.5;60.0]	36.0 [31.0;49.5]	30.0 [22.0;44.0]	< 0.001
OASIS	34.0 [28.0;42.0]	37.0 [30.0;45.0]	33.0 [28.0;38.0]	33.0 [26.0;38.0]	0.004
LODS	6.00 [4.00;9.00]	8.00 [5.75;10.2]	7.00 [4.00;9.00]	5.00 [3.00;7.00]	< 0.001
SOFA	7.00 [4.00;11.0]	10.0 [6.00;13.0]	7.00 [4.00;10.0]	5.00 [3.00;8.00]	< 0.001
CHARLSON	4.00 [2.00;6.00]	5.00 [2.00;7.00]	4.00 [2.00;7.00]	4.00 [2.50;5.00]	0.526
Heart failure	64 (28.8%)	27 (33.8%)	25 (35.2%)	12 (16.9%)	0.026
Hypertension	104 (46.8%)	33 (41.2%)	35 (49.3%)	36 (50.7%)	0.449
Atrial fibrillation	15 (6.76%)	6 (7.50%)	4 (5.63%)	5 (7.04%)	0.945
Diabetes	59 (26.6%)	23 (28.7%)	19 (26.8%)	17 (23.9%)	0.800
CKD	44 (19.8%)	18 (22.5%)	19 (26.8%)	7 (9.86%)	0.031
CAD	45 (20.3%)	13 (16.2%)	23 (32.4%)	9 (12.7%)	0.007
Liver cirrhosis	25 (11.3%)	22 (27.5%)	1 (1.41%)	2 (2.82%)	< 0.001
MAP (/mmHg)	57.0 [50.0;64.0]	55.0 [47.0;61.0]	57.0 [50.5;64.0]	59.0 [52.0;65.5]	0.044
SBP (/mmHg)	83.0 [73.0;94.0]	80.0 [70.8;88.2]	84.0 [73.0;94.0]	88.0 [78.5;102]	0.001
DBP (/mmHg)	46.5 [39.0;52.0]	43.0 [34.8;49.0]	48.0 [39.5;52.0]	48.0 [42.0;54.0]	0.001
Temperature (℃)	37.4 [37.1;38.2]	37.2 [37.0;37.8]	37.7 [37.2;38.7]	37.7 [37.2;38.2]	0.017
Heart rate (bpm)	112 [97.2;131]	115 [101;137]	113 [100;130]	108 [92.5;120]	0.020
Respiratory rate (/min)	29.0 [25.0;34.0]	30.5 [25.8;34.2]	29.0 [26.0;35.0]	28.0 [25.0;32.0]	0.264
Spo2 (%)	92.0 [90.0;95.0]	92.0 [87.0;94.0]	93.0 [91.0;95.0]	93.0 [91.0;95.5]	0.004
PH	7.30 [7.20;7.38]	7.29 [7.13;7.36]	7.30 [7.20;7.38]	7.34 [7.28;7.40]	0.025
PO2 (/mmHg)	80.0 [62.0;112]	79.0 [64.0;107]	75.0 [60.0;99.5]	85.0 [67.5;134]	0.263
PCO2 (/mmHg)	44.5 [37.2;53.8]	43.0 [36.5;53.0]	47.0 [41.0;57.0]	43.0 [35.2;50.8]	0.099
Bicarbonate (mmol/L)	19.4 (5.18)	17.4 (5.02)	19.9 (5.53)	21.1 (4.16)	< 0.001
WBC (× 10⁹/L)​​	15.1 [10.7;21.3]	17.4 [13.1;25.6]	13.3 [8.60;20.0]	13.4 [10.8;18.2]	0.001
RBC (× 10^12^/L)	3.70 [3.09;4.35]	3.36 [3.00;4.14]	3.63 [3.07;4.41]	3.96 [3.39;4.38]	0.015
Platelet (× 10⁹/L)​​	148 [77.8;244]	106 [62.2;165]	167 [92.5;280]	170 [106;281]	0.001
Hemoglobin (g/dL)	9.34 (2.34)	8.66 (2.08)	9.31 (2.12)	10.1 (2.60)	0.001
Lactate (mmol/L)	3.00 [1.70;5.85]	4.00 [2.82;8.73]	2.40 [1.50;5.35]	2.10 [1.48;3.52]	< 0.001
Creatinine (mg/dL)	1.30 [0.90;2.60]	2.15 [1.28;3.30]	1.30 [1.00;2.60]	0.90 [0.80;1.30]	< 0.001
BUN (mmol/L)	24.0 [16.0;43.0]	35.0 [19.8;59.5]	26.0 [17.5;42.0]	17.0 [13.0;26.0]	< 0.001
ALT (U/L)	53.0 [22.0;182]	76.5 [27.5;356]	48.5 [23.5;201]	29.0 [18.0;81.0]	0.021
AST (U/L)	79.0 [41.0;268]	119 [56.0;932]	87.5 [39.5;268]	59.0 [32.0;103]	0.005
TBIL (mg/dL)	1.20 [0.60;2.70]	2.20 [1.10;5.55]	0.80 [0.50;1.90]	0.70 [0.40;1.20]	< 0.001
ALB (g/dL)	2.60 [2.10;3.30]	2.35 [2.00;3.10]	2.35 [2.02;3.00]	3.15 [2.68;3.62]	0.001
Calcium (mg/dL)	7.80 [7.30;8.40]	7.55 [6.90;8.30]	7.80 [7.15;8.35]	8.15 [7.70;8.50]	0.002
Chloride (mmol/L)	101 (7.03)	98.0 (6.92)	101 (7.36)	103 (5.72)	< 0.001
Sodium (mmol/L)	135 [131;138]	133 [129;137]	135 [132;137]	137 [134;140]	< 0.001
Potassium (mmol/L)	4.50 [4.10;5.05]	4.70 [4.20;5.43]	4.50 [4.00;5.10]	4.20 [4.00;4.70]	0.005
Glucose (mg/dL)	172 [136;255]	176 [149;246]	195 [138;286]	154 [125;210]	0.027
INR	1.60 [1.30;2.05]	2.00 [1.60;3.12]	1.60 [1.40;1.90]	1.30 [1.20;1.55]	< 0.001
PT (s)	17.6 [14.6;22.0]	21.4 [17.8;34.4]	17.3 [14.8;20.3]	14.8 [13.4;17.3]	< 0.001
APTT (s)	40.0 [31.1;69.5]	54.6 [36.7;105]	43.8 [33.8;77.4]	31.7 [27.7;39.7]	< 0.001
28-day death	54 (24.3%)	34 (42.5%)	9 (12.7%)	11 (15.5%)	< 0.001
ICU death	47 (21.2%)	32 (40.0%)	9 (12.7%)	6 (8.5%)	< 0.001
DIC, events	37 (16.7%)	27 (33.8%)	7 (9.9%)	3 (4.2%)	< 0.001
AKI, events	200 (90.1%)	76 (95.0%)	65 (91.5%)	59 (83.1%)	0.045

### Statistical analysis

Continuous variables are presented as mean ± standard deviation (SD), and categorical variables as counts (percentages). Group comparisons for continuous and categorical variables were performed using the Kruskal–Wallis rank-sum test and the Chi-square test, respectively. Time-to-event analysis was conducted to assess associations between variables and all-cause mortality. The proportional hazards assumption was verified for each variable using Schoenfeld residuals. Univariable and multivariable Cox proportional hazards regression models were employed to identify factors independently associated with mortality.

For preliminary and descriptive purposes, AT activity was categorized into three groups based on tertiles of the study population: T1 (< 46%), T2 (46–73%), and T3 (> 73%). This categorization was used to visualize broad trends and for baseline comparisons in Table 1. Multivariable models were adjusted for demographic factors including age (modeled as a continuous variable), race and ethnicity (White, Black, Asian, Other), sex, the Charlson Comorbidity Index (CCI), and the Sequential Organ Failure Assessment (SOFA) score. To account for multiple comparisons, the Benjamini–Hochberg procedure was applied to control the false discovery rate (FDR). For trend analysis based on tertiles, the median value of each AT activity tertile was treated as a continuous variable in the regression model. Multicollinearity in the multivariable Cox model was assessed using variance inflation factors (VIF), with a VIF < 10 indicating acceptable levels of collinearity.

The primary analysis to characterize the dose–response relationship and to identify prognostic thresholds was performed by incorporating AT activity as a continuous variable using restricted cubic splines (RCS). Cutoff values associated with mortality risk were derived from the RCS curve. Based on the identified cutoff, the study cohort was stratified into distinct risk groups for subsequent comparison of primary and secondary outcomes.

Stratified analyses were performed according to key demographic characteristics and the following comorbidities: atrial fibrillation, coronary artery disease, heart failure, hypertension, diabetes, liver cirrhosis, and chronic kidney disease. Multiplicative interaction terms between subgroups and AT activity were included in the models to evaluate potential effect modification. For any subgroup demonstrating a statistically significant interaction, we planned to perform a dedicated RCS analysis within that subgroup to characterize its specific dose–response relationship with mortality and to identify a subgroup-specific risk threshold, if applicable. Subsequently, patients within that subgroup would be stratified based on the identified threshold for comparative analyses of baseline characteristics and clinical outcomes, analogous to the approach used for the overall cohort. A p-value < 0.05 was considered statistically significant. All statistical analyses were performed using R software (version 4.5.0).

## Results

### Baseline

The baseline characteristics of the 222 enrolled sepsis patients are summarized in Table 1. Patients were stratified into three groups based on AT activity tertiles: T1 (< 46%), T2 (46–73%), and T3 (> 73%). Comparative analyses revealed statistically significant differences across these groups for numerous parameters reflecting disease severity and organ dysfunction. Specifically, all five illness severity scores—APSIII, SAPSII, LODS, SOFA, and OASIS—were significantly elevated in the T1 group compared to the T3 group. The prevalence of several comorbidities, including heart failure, CKD, CAD, and liver cirrhosis, also differed significantly among the tertiles. Key laboratory markers, such as lactate, creatinine, TBIL, INR, PT, and APTT, were most markedly abnormal in the T1 group. Consequently, critical outcomes, including ICU mortality, 28-day mortality, DIC, and AKI, occurred significantly more frequently in the T1 group.

### Association between AT activity and 28-day mortality using tertile categories

As shown in Table 2, a clear inverse association was observed between AT activity and the risk of 28-day mortality in septic patients. In the unadjusted model (Model 1), compared to patients in the lowest tertile (T1, < 46%), those in the middle (T2, 46–73%) and highest (T3, > 73%) tertiles exhibited significantly reduced mortality risks, with hazard ratios (HRs) of 0.26 (95% CI 0.12–0.54) and 0.31 (95% CI: 0.16–0.62), respectively. This protective association persisted after adjustment for demographics and comorbidities. After adjusting for age, sex, race/ethnicity, and the Charlson Comorbidity Index (Model 2), the HRs for T2 and T3 were 0.28 (95% CI 0.13–0.60) and 0.28 (95% CI 0.14–0.57). Furthermore, when analyzed as a continuous variable, each 1% increase in AT activity was associated with a 2% reduction in mortality risk in both Model 1 and Model 2 (HR 0.98, 95% CI 0.97–0.99). To address potential confounding by illness severity, we further adjusted for the Sequential Organ Failure Assessment (SOFA) score in Model 3. After this adjustment, the inverse association between AT activity and 28-day mortality remained statistically significant. The HRs for T2 and T3 were 0.34 (95% CI 0.16–0.72) and 0.47 (95% CI 0.23–0.98), respectively. When analyzed as a continuous variable, each 1% increase in AT activity remained associated with a 2% reduction in mortality risk (HR 0.98, 95% CI 0.97–0.99). The p-value for trend across tertiles in Model 3 was 0.004.

**Table 2:** Multivariable Cox proportional hazards analysis

Models	Antithrombin			Antithrombin Increase 1 percent	p for trend
	T1(< 46%)	T2(46–73%)	T3(> 73%)		
28-day mortality					
Model 1 HR( 95%CI)	ref	0.26(0.12,0.54)	0.31(0.16,0.62)	0.98(0.97,0.99)	< 0.001
Model 2 HR( 95%CI)	ref	0.28(0.13,0.60)	0.28(0.14,0.57)	0.98(0.97,0.99)	< 0.001
Model 3 HR( 95%CI)	ref	0.34(0.16,0.72)	0.47(0.23,0.98)	0.98(0.97,0.99)	0.004

Model 1: non-adjusted

Model 2: adjusted for age, sex, race/ethnicity, CCI

Model 3: adjusted for age, sex, race/ethnicity, CCI, SOFA

### Dose–response relationship and prognostic threshold identification using RCS

**Fig. 2:**
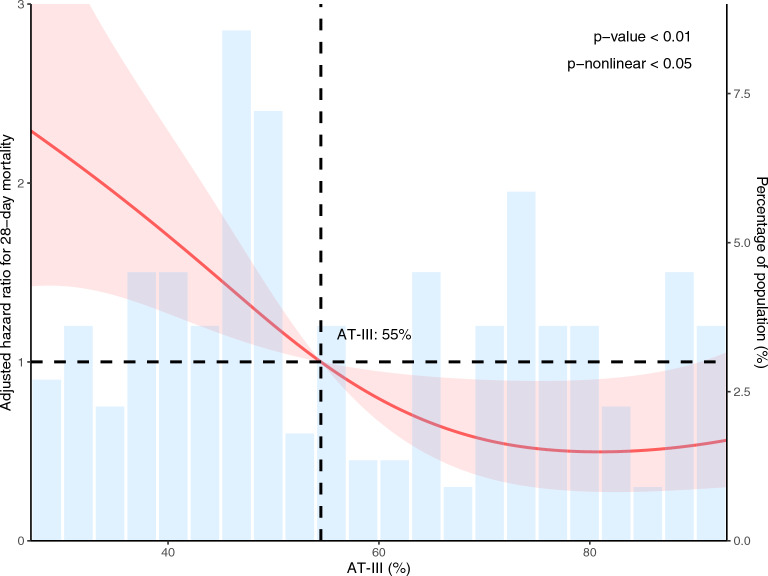
RCS analysis of the association between AT activity and 28-day mortality risk. *RCS* restricted cubic splines, *HR* hazard ratio, *CI* confidence interval.

The dose–response relationship between AT activity and 28-day mortality was further elucidated using RCS analysis. As shown in Fig. 2, the analysis revealed a statistically significant non-linear relationship (p for non-linear < 0.05), with an overall p-value of < 0.01. The RCS curve indicated that mortality risk increased markedly when AT activity fell below approximately 55%. Within this lower activity range, the adjusted hazard ratio rose steeply with decreasing AT levels, identifying a zone of substantially elevated risk. Above approximately 55%, the mortality risk plateaued at a lower level. The population distribution, as shown by the histogram, indicated that a considerable proportion of patients had AT levels within this higher-risk range.

### Comparison between low and high AT groups

**Fig. 3:**
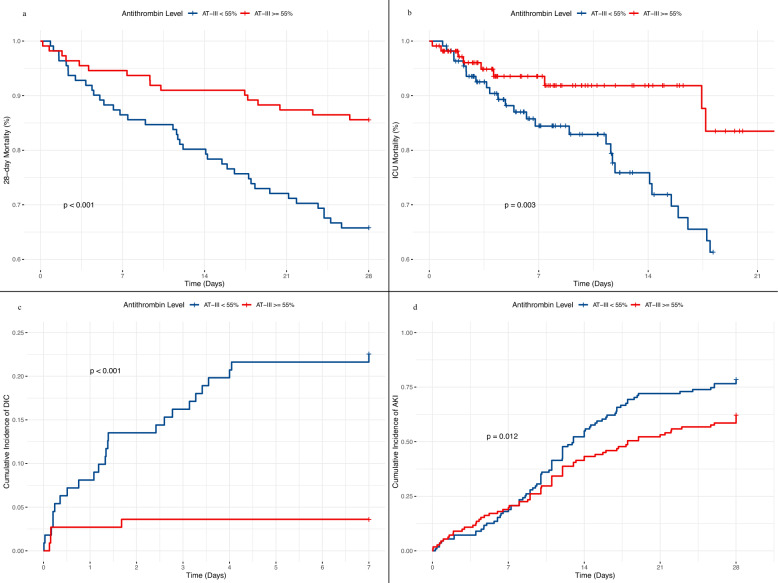
Kaplan–Meier curves comparing clinical outcomes between septic patients stratified by AT activity levels. **a** 28-day mortality, **b** ICU mortality, **c** cumulative incidence of DIC, **d** cumulative incidence of AKI

To evaluate the clinical profile associated with the higher-risk zone indicated by the RCS analysis, the cohort was stratified into two groups based on an AT activity threshold of approximately 55%: a low AT group (< 55%) and a high AT group (≥ 55%). Comparative analyses of baseline characteristics and clinical outcomes between these groups are summarized in Table S1. The low AT group demonstrated significantly greater disease severity, as indicated by elevated scores across all assessed illness severity scales (APSIII, SAPSII, OASIS, LODS, and SOFA; p < 0.001 for all). Furthermore, this group exhibited widespread laboratory evidence of multi-organ dysfunction, including impaired coagulation parameters such as INR, PT, and APTT (p < 0.05). Consequently, patients in the low AT group experienced significantly poorer clinical outcomes, with higher incidences of 28-day mortality (34.2% vs. 14.4%, p = 0.001), ICU mortality (33.3% vs. 9.0%, p < 0.001), DIC (22.5% vs. 3.6%, p < 0.001), and AKI (78.4% vs. 62.2%, p = 0.013). These stark outcome disparities were visually corroborated by Kaplan–Meier curves (Fig. 3), which illustrated pronounced separation in event-free probability between the two groups across all evaluated endpoints.

### Subgroup analysis

Subgroup analyses were conducted to evaluate the consistency of the association between AT activity and 28-day mortality across key patient characteristics (Figure S1). The protective effect of higher AT activity was generally consistent across most predefined subgroups, including those defined by age, sex, and several major comorbidities. A statistically significant interaction was observed for hypertension status (p for interaction = 0.007). Specifically, the inverse association between AT activity and 28-day mortality risk was significantly more pronounced in patients with a history of hypertension compared to those without. No other significant interactions were identified.

### Dose–response relationship in hypertensive subgroup

**Fig. 4:**
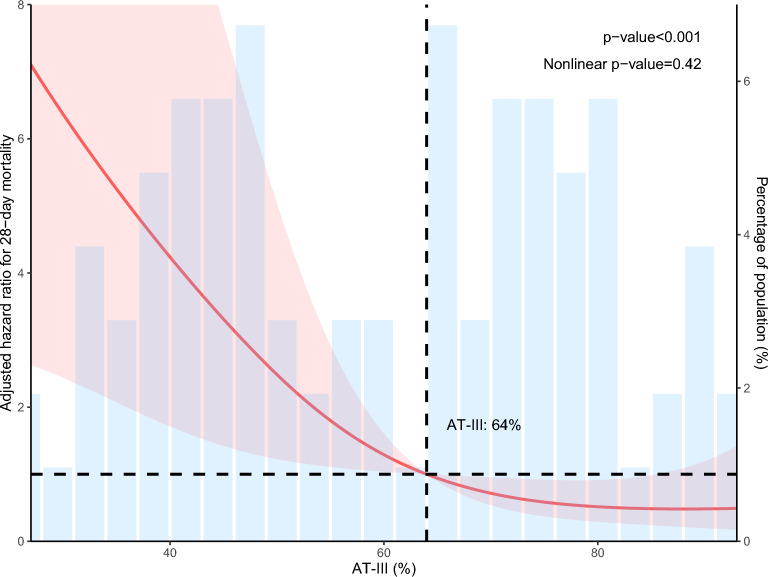
RCS analysis of the association between AT activity and 28-day mortality risk in the hypertensive subgroup. *RCS* restricted cubic splines, *HR* hazard ratio, *CI* confidence interval

RCS analysis confirmed a statistically significant association between AT activity and 28-day mortality in the hypertensive subgroup (overall p < 0.001)（Fig. 4). The curve indicated a marked increase in mortality risk when AT activity fell below approximately 64%. Based on this threshold, the hypertensive cohort was stratified into two groups for subsequent comparison: a lower-activity group (< 64%) and a higher-activity group (≥ 64%). Comparative analyses of baseline characteristics and clinical outcomes between these groups are presented in Table S2. The lower AT group exhibited significantly greater disease severity, as evidenced by elevated scores across key illness severity scales, including APSIII (66.4 vs. 48.5, p < 0.001), SAPSII (47.3 vs. 32.8, p < 0.001), OASIS (36.2 vs. 32.1, p = 0.014), LODS (7.52 vs. 5.52, p = 0.002), and SOFA (8.71 vs. 5.60, p < 0.001). This group also demonstrated marked laboratory evidence of multi-organ dysfunction, such as significantly higher lactate (6.07 vs. 2.52 mmol/L, p < 0.001), creatinine (2.93 vs. 1.86 mg/dL, p = 0.029), and impaired coagulation parameters (e.g., INR 2.63 vs. 1.62, p = 0.003). Consequently, the lower AT group experienced significantly poorer outcomes, with higher incidences of 28-day mortality (42.3% vs. 9.62%, p < 0.001), ICU mortality (38.5% vs. 5.77%, p < 0.001), and DIC (19.2% vs. 1.92%, p = 0.011). In contrast, the incidence of AKI, while numerically higher in the lower AT group (78.8% vs. 63.5%), did not reach statistical significance (p = 0.130). The Kaplan–Meier curves visually confirmed these outcome disparities, demonstrating a distinct separation in event-free probability between the two groups for the endpoints of 28-day mortality, ICU mortality, and DIC

(Fig. 5).

**Fig. 5:**
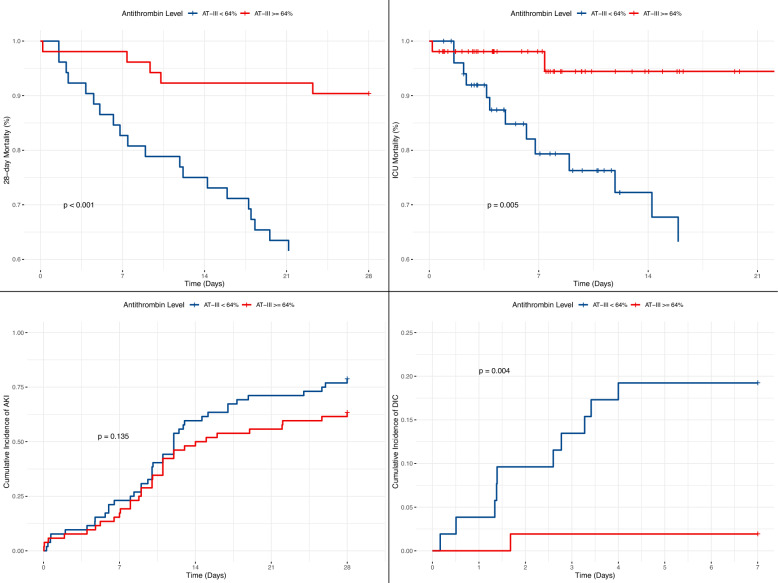
Kaplan–Meier curves comparing clinical outcomes between septic patients stratified by AT activity levels in the hypertensive subgroup. **a** 28-day mortality, **b** ICU mortality, **c** cumulative incidence of DIC, **d** cumulative incidence of AKI

## Discussion

To investigate the association between AT deficiency and adverse clinical outcomes in sepsis, we analyzed septic patients from the MIMIC-IV and MIMIC-III databases. This study identifies potential prognostic thresholds for AT activity—approximately 55% in the overall cohort and 64% in hypertensive patients—and characterizes their relationship with key endpoints, including 28-day mortality, ICU mortality, DIC, and AKI. Our results show that AT activity below these levels is strongly associated with poorer outcomes. Subgroup analysis indicated that this association was particularly pronounced in septic patients with hypertension. Collectively, these findings highlight the role of AT activity in risk stratification and underscore the value of these thresholds for defining high-risk phenotypes, which may inform future research on personalized management strategies.

Previous studies have established a significant correlation between AT levels and patient outcomes across various critical care settings. For instance, Farrell et al. demonstrated in trauma patients that AT deficiency was associated with reduced durations of ventilator-free, hospital-free, and ICU-free periods, alongside an elevated risk of progressive intracranial hemorrhage and thrombotic complications, suggesting AT as a potential biomarker for disease severity [26]. Similarly, in sepsis, Tagami et al. observed that abdominal sepsis patients with low AT levels had higher mortality rates, and AT supplementation appeared to improve outcomes based on propensity score-matched analyses, indicating a beneficial effect in this subgroup [27]. Recent investigations have further refined this understanding. Yarimizu et al. confirmed that early measured AT activity independently predicted ICU mortality, particularly showing higher specificity for predicting survival in patients with an intermediate risk of death [28]. In contrast, Zeng et al. reported that AT had limited predictive value for 28-day mortality in sepsis patients, although it might be associated with early DIC development [29]. In another study, Li et al. reported that AT levels measured within the first 12 h did not significantly predict outcomes, highlighting the potential importance of the measurement window [30].

The apparent inconsistencies among studies likely arise from several key methodological variations, such as whether studies aimed to identify prognostically discriminatory activity thresholds or accounted for population heterogeneity. Our study contributes to this field by quantitatively modeling the association between AT activity and mortality using RCS, which led to the identification of exploratory thresholds at approximately 55% (overall cohort) and 64% (hypertensive subgroup). These data-informed estimates may help reconcile disparate findings in the literature, as the mortality risk appears to increase markedly below these levels. This approach aligns with the integrative risk-stratification concept proposed by Iba et al. [31]. Their primary utility lies in defining a high-risk phenotype for prognostic enrichment in subsequent research, particularly for investigating potential targeted strategies in patients with activity below these levels.

AT is a central serine protease inhibitor with dual anticoagulant and anti-inflammatory functions. Under septic conditions, its deficiency becomes a critical node linking coagulopathy and immune dysregulation, disrupting natural anticoagulant mechanisms and promoting the microvascular thrombosis characteristic of DIC [32, 33]. Concurrently, diminished anti-inflammatory capacity exacerbates neutrophil overactivation and cytokine release, amplifying endothelial injury and organ dysfunction [14]. This dual-system failure establishes AT deficiency as a marker of endogenous protective collapse.

A significant pathophysiological linkage exists between low AT levels and the high incidence of DIC and AKI in septic patients, arising from the interplay of coagulation dysregulation, inflammatory amplification, and microcirculatory failure [14, 34]. Profound AT deficiency compromises crucial host defense mechanisms, creating a permissive environment for thrombotic and inflammatory organ injury. The loss of its anticoagulant function leads to insufficient inhibition of key serine proteases, notably thrombin and factor Xa, resulting in uncontrolled coagulation cascade activation, widespread fibrin deposition, and platelet consumption [35]. Concurrently, diminished anti-inflammatory capacity promotes neutrophil overactivation and neutrophil extracellular trap (NET) release, which further exacerbates the prothrombotic state [36]. This interaction establishes a vicious cycle of thrombo-inflammation, wherein coagulation and inflammation mutually reinforce each other, ultimately culminating in extensive microvascular thrombosis and impaired tissue perfusion. Furthermore, the development of AKI in this context is a direct consequence of microcirculatory failure orchestrated by AT deficiency. The kidneys, with their dense capillary networks, are highly vulnerable to micro-thrombosis formation within glomerular and peritubular capillaries, leading to renal ischemia and functional impairment [37]. Concurrent degradation of the endothelial glycocalyx increases vascular permeability and exacerbates endothelial injury, further compromising renal perfusion. The loss of AT-mediated suppression of leukocyte activation also renders renal tubular cells more susceptible to direct inflammatory attack. The confluence of ischemic injury from microthrombi and direct inflammatory damage explains the strong association between low AT levels and AKI incidence [38]. Clinically, the consumption of AT during sepsis potentially aggravates the imbalance between coagulation and inflammation systems, thereby affecting microcirculation and organ function. Our findings provide a novel perspective for subsequent research on the prognostic value of AT in septic patients, highlighting its potential role in risk stratification and guiding future therapeutic strategies.

In patients with a history of hypertension who develop sepsis, the association between AT deficiency and adverse outcomes may stem from the interaction between hypertension-induced endothelial dysfunction and the physiological functions of AT. Hypertension is characterized by chronic endothelial pathology, including persistent low-grade inflammation, increased oxidative stress, and glycocalyx damage, collectively reducing endothelial functional reserve and increasing microvascular vulnerability [39]. When sepsis occurs, this already compromised endothelium faces a dual assault from acute inflammatory and coagulation responses [40]. In the setting of pre-existing endothelial damage, the endothelium becomes increasingly reliant on AT-mediated protection. The persistent activation of the renin–angiotensin system (RAS) in hypertension further aggravates this dependency; angiotensin II-induced oxidative stress and pro-inflammatory responses exacerbate endothelial dysfunction, effects that may be partially counteracted by the anti-inflammatory properties of AT [41, 42]. This may explain why a higher threshold of AT activity (≥ 64%) is critical for a favorable prognosis in the hypertensive subgroup. Alterations in the expression of endothelial adhesion molecules in hypertension may also increase sensitivity to AT-mediated protection. When AT levels are insufficient, hypertension-associated increases in vascular stiffness and endothelial permeability likely accelerate microcirculatory failure, leading to impaired organ perfusion [43]. These mechanisms collectively establish septic patients with hypertension as a distinct pathophysiological subtype, whose endothelial vulnerability reduces tolerance to AT deficiency. This understanding not only explains the observed clinical associations, but also provides a rationale for targeted therapeutic strategies in this specific population. Future research should focus on elucidating the molecular crosstalk between hypertension and sepsis-induced endothelial injury and defining the regulatory role of AT within this interplay.

This study has several important limitations that should be considered when interpreting the results. First, the inherent limitations of observational data, combined with the moderate sample size of this cohort, preclude definitive causal inference and limit the applicability of methods specifically designed to address baseline imbalances. Therefore, to address the central concern of confounding by disease severity as rigorously as possible, we explicitly adjusted for the SOFA score in our multivariable analysis. Although the significant association between low AT activity and adverse outcomes persisted after this adjustment, which suggests its prognostic value may extend beyond baseline organ failure, it remains challenging to fully disentangle whether AT deficiency is an independent driver of poor outcomes or a highly sensitive integrative biomarker of the overall pathological burden. Consequently, the observed association is most appropriately interpreted as identifying a high-risk integrative clinical phenotype; its independence from all severity-related confounders requires validation in larger, prospective studies that can employ more robust methods for confounding control. Second, the risk thresholds identified through RCS analysis are exploratory in nature. RCS is valuable for visualizing non-linear relationships and highlighting ranges of increased risk; however, the specific numerical thresholds should be regarded as data-informed estimates useful for risk stratification and hypothesis generation rather than as definitive biological or clinical cut-points. Their stability and clinical relevance require validation in independent prospective cohorts. Furthermore, the subgroup-specific threshold (64%) observed in hypertensive patients is particularly exploratory. This interaction was identified without adjustment for multiple comparisons, and the subgroup sample size is limited; therefore, this finding should be interpreted as generating a focused hypothesis for future validation rather than as a definitive clinical cutoff. Third, the interpretation of multiple clinical outcomes requires caution regarding the risk of false-positive findings. While the primary analysis focused on 28-day mortality, the assessment of secondary endpoints (ICU mortality, DIC, and AKI) involved multiple comparisons. Although we applied false discovery rate (FDR) control where appropriate, the results for these secondary endpoints should be viewed as supporting the characterization of a high-risk phenotype rather than as independently conclusive. The consistent direction of risk elevation across all endpoints, however, strengthens the overall conclusion. Fourth, this study is constrained by a lack of longitudinal and therapeutic data in the database. Our analysis was limited to the initial AT level; the absence of repeated measurements prevented assessment of its dynamic changes or the prognostic value of achieving a specific post-treatment activity target. Moreover, detailed information on the administration of therapies that influence AT levels was not available, precluding any evaluation of supplementation therapy on outcomes. Fifth, as a retrospective analysis based on data from a single center, our findings may be influenced by unmeasured confounders and local practice patterns, which could affect generalizability. These constraints collectively underscore the necessity for future prospective, multicenter studies that incorporate routine AT monitoring, detailed treatment documentation, and validation of the proposed risk thresholds to confirm our findings and further elucidate the dynamic role of AT in sepsis management.

## Conclusion

This study demonstrates that reduced AT activity is significantly associated with higher mortality and organ dysfunction in sepsis, with risk thresholds observed at approximately 55% for the overall cohort and 64% among hypertensive patients. Below these levels, clinical risk escalated substantially, marked by a significantly increased incidence of DIC and AKI in addition to elevated mortality. These findings support the role of AT activity as a prognostic biomarker for risk stratification and highlight its potential to inform future targeted management strategies for high-risk septic patients, particularly those with comorbidities such as hypertension.

## Supplementary Information

Supplementary file 1. Figure S1. Subgroup analysis of the association between AT activity and 28-day mortality in septic patients.​​. HR, hazard ratio; CI, confidence interval.

Supplementary file 2.

Supplementary file 3.

## Data Availability

The datasets generated and analyzed during the current study are available from the corresponding author on reasonable request.

## Abbreviations

AT: Antithrombin
SIC: Sepsis-induced coagulopathy
DIC: Disseminated intravascular coagulation
AKI: Acute kidney injury
RAS: Renin–angiotensin system
NETs: Neutrophil extracellular traps
MIMIC-IV: Medical Information Mart for Intensive Care IV
MIMIC-III: Medical Information Mart for Intensive Care III
BIDMC: Beth Israel Deaconess Medical Center
ICU: Intensive care unit
IRB: Institutional Review Board
MIT: Massachusetts Institute of Technology
APSIII: Acute Physiology Score III
SAPSII: Simplified Acute Physiology Score II
SOFA: Sequential Organ Failure Assessment
LODS: Logistic Organ Dysfunction System
OASIS: Oxford Acute Severity of Illness Score
CCI: Charlson Comorbidity Index
KDIGO: Kidney Disease: Improving Global Outcomes
ISTH: International Society on Thrombosis and Haemostasis
RCS: Restricted cubic splines
HR: Hazard ratio
CI: Confidence interval
SD: Standard deviation
FDR: False discovery rate
VIF: Variance inflation factor
INR: International normalized ratio
PT: Prothrombin time
APTT: Activated partial thromboplastin time
BMI: Body mass index
MAP: Mean arterial pressure
