# Supplementary figures and images for: Association between antithrombin levels and prognosis in patients with sepsis: a retrospective cohort study based on the MIMIC-IV and MIMIC-III databases
Source: J Intensive Care. 2026 Feb 2;14:24. doi: 10.1186/s40560-026-00862-x (PMC12952102; doi:10.1186/s40560-026-00862-x)

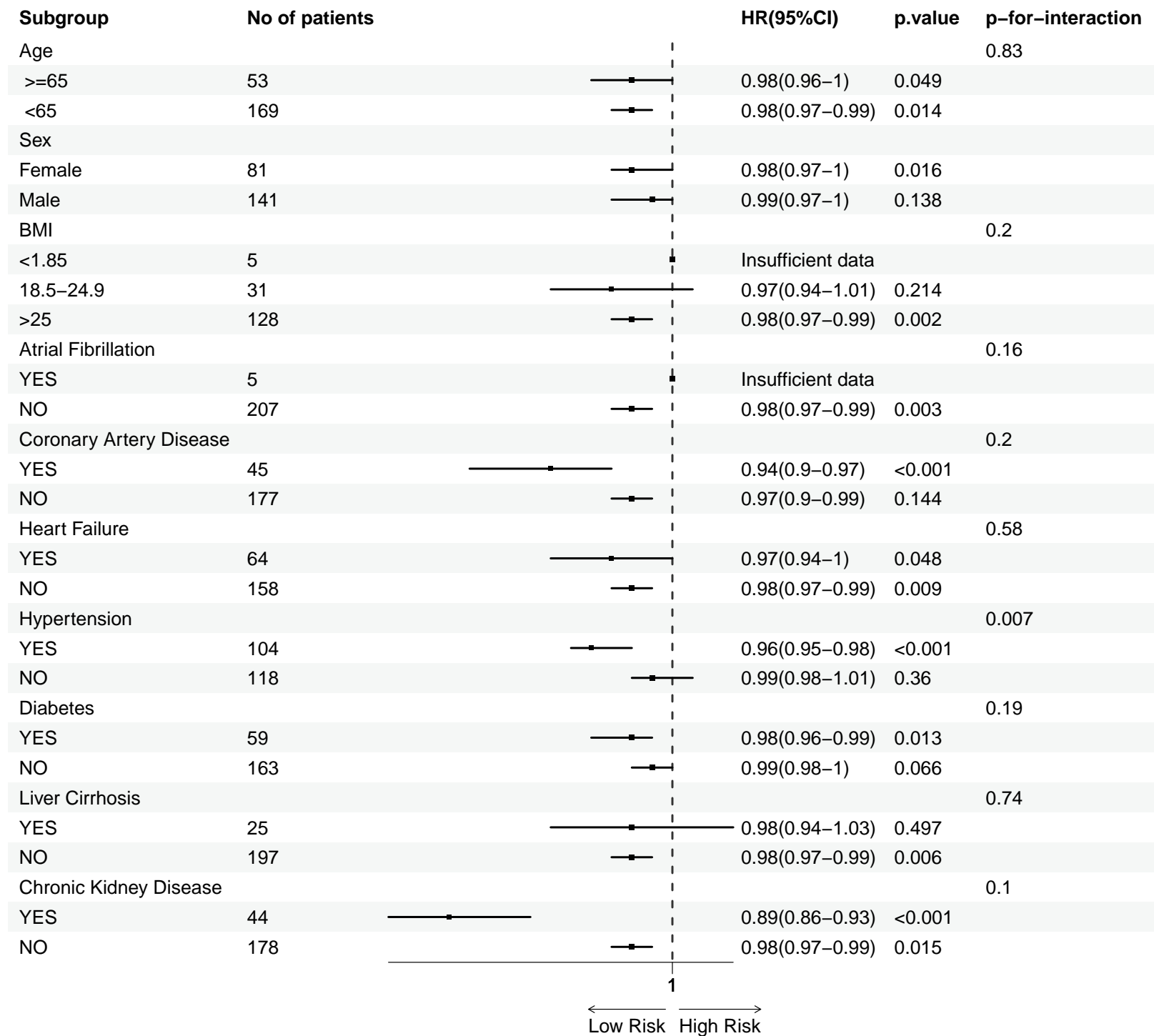

Supplement: Supplementary file 1 — Figure S1. Subgroup analysis of the association between AT activity and 28-day mortality in septic patients.​​. HR, hazard ratio; CI, confidence interval. [file 40560_2026_862_MOESM1_ESM.pdf]
