# Supplementary material for: Association between antithrombin levels and prognosis in patients with sepsis: a retrospective cohort study based on the MIMIC-IV and MIMIC-III databases
Source: J Intensive Care. 2026 Feb 2;14:24. doi: 10.1186/s40560-026-00862-x (PMC12952102; doi:10.1186/s40560-026-00862-x)
Supplement: Supplementary file 2 [file 40560_2026_862_MOESM2_ESM.docx]

**Table S1.** Characteristics and outcomes of participants stratified by AT-III activity levels.​​

| **Characteristic** | **Overall** | **AT-III < 55%** | **AT-III ​≥​ 55%** | **p-value** |
| --- | --- | --- | --- | --- |
|  | ***N=222*** | ***N=111*** | ***N=111*** |  |
| Antithrombin (%) | 59.7 (27.6) | 37.1 (12.3) | 82.3 (18.7) | <0.001 |
| Age (years) | 52.7 (16.4) | 53.6 (17.3) | 51.7 (15.4) | 0.409 |
| Sex: |  |  |  | 1.000 |
| Female | 81 (36.5%) | 41 (36.9%) | 40 (36.0%) |  |
| Male | 141 (63.5%) | 70 (63.1%) | 71 (64.0%) |  |
| Race: |  |  |  | 0.046 |
| White | 111 (50.0%) | 64 (57.7%) | 47 (42.3%) |  |
| Black | 27 (12.2%) | 8 (7.21%) | 19 (17.1%) |  |
| Other | 84 (37.8%) | 39 (35.1%) | 45 (40.5%) |  |
| Weigh (kg) | 89.6 (22.4) | 88.5 (19.8) | 90.6 (24.7) | 0.474 |
| Height (cm) | 173 (9.37) | 172 (9.09) | 173 (9.74) | 0.564 |
| BMI (kg/m^2^) | 30.3 (7.60) | 29.8 (6.41) | 30.8 (8.89) | 0.429 |
| APSIII | 60.9 (25.7) | 70.9 (25.3) | 50.9 (22.1) | <0.001 |
| SAPSII | 41.0 (16.7) | 46.7 (16.9) | 35.4 (14.5) | <0.001 |
| OASIS | 34.7 (8.86) | 36.8 (9.18) | 32.6 (8.01) | <0.001 |
| LODS | 6.74 (3.38) | 7.73 (3.41) | 5.76 (3.06) | <0.001 |
| SOFA | 7.62 (4.21) | 9.09 (4.32) | 6.15 (3.53) | <0.001 |
| CHARLSON | 4.42 (2.71) | 4.48 (3.00) | 4.36 (2.40) | 0.749 |
| Heart Failure | 64 (28.8%) | 36 (32.4%) | 28 (25.2%) | 0.300 |
| Hypertension | 104 (46.8%) | 44 (39.6%) | 60 (54.1%) | 0.044 |
| Atrial Fibrillation | 15 (6.76%) | 8 (7.21%) | 7 (6.31%) | 1.000 |
| Diabetes | 59 (26.6%) | 29 (26.1%) | 30 (27.0%) | 1.000 |
| CKD | 44 (19.8%) | 22 (19.8%) | 22 (19.8%) | 1.000 |
| CAD | 45 (20.3%) | 22 (19.8%) | 23 (20.7%) | 1.000 |
| Liver Cirrhosis | 25 (11.3%) | 22 (19.8%) | 3 (2.70%) | <0.001 |
| MAP (/mmHg) | 54.8 (16.4) | 51.5 (17.3) | 58.1 (14.8) | 0.002 |
| SBP (/mmHg) | 84.0 (18.6) | 79.5 (17.2) | 88.6 (18.9) | <0.001 |
| DBP (/mmHg) | 45.2 (11.5) | 42.3 (11.7) | 48.0 (10.6) | <0.001 |
| Temperature (℃) | 114 (23.4) | 120 (24.4) | 109 (21.2) | 0.001 |
| Heart Rate (bpm) | 37.7 (0.92) | 37.7 (0.93) | 37.8 (0.91) | 0.183 |
| Respiratory Rate (/min) | 30.0 (7.45) | 30.9 (7.95) | 29.0 (6.84) | 0.066 |
| Spo2 (%) | 90.8 (8.81) | 89.4 (11.2) | 92.2 (5.14) | 0.019 |
| PH | 7.28 (0.13) | 7.26 (0.14) | 7.31 (0.11) | 0.007 |
| PO2 (/mmHg) | 97.2 (61.3) | 95.1 (64.7) | 99.6 (57.6) | 0.651 |
| PCO2 (/mmHg) | 47.4 (16.1) | 48.4 (18.1) | 46.4 (13.5) | 0.438 |
| Bicarbonate (mmol/L) | 19.4 (5.18) | 17.9 (5.29) | 20.8 (4.65) | <0.001 |
| WBC (×10⁹/L)​​ | 19.2 (27.4) | 20.1 (13.5) | 18.3 (36.6) | 0.624 |
| RBC (×10¹²/L) | 3.71 (0.78) | 3.57 (0.80) | 3.85 (0.74) | 0.007 |
| Platelet (×10⁹/L)​​ | 172 (122) | 148 (120) | 197 (119) | 0.003 |
| Hemoglobin (g/dL) | 9.34 (2.34) | 8.67 (2.01) | 10.0 (2.45) | <0.001 |
| Lactate (mmol/L) | 4.73 (4.39) | 6.15 (4.85) | 2.89 (2.81) | <0.001 |
| Creatinine (mg/dL) | 2.00 (1.98) | 2.47 (2.28) | 1.53 (1.47) | <0.001 |
| BUN (mmol/L) | 33.0 (24.7) | 38.2 (25.8) | 27.6 (22.4) | 0.001 |
| ALT (U/L) | 567 (1750) | 859 (2223) | 176 (555) | 0.004 |
| AST (U/L) | 1013 (2889) | 1536 (3635) | 312 (1019) | 0.002 |
| TBIL (mg/dL) | 2.88 (4.81) | 3.80 (5.32) | 1.68 (3.74) | 0.002 |
| ALB (g/dL) | 2.72 (0.76) | 2.54 (0.76) | 2.99 (0.67) | 0.002 |
| Calcium (mg/dL) | 7.81 (1.00) | 7.56 (1.09) | 8.08 (0.82) | <0.001 |
| Chloride (mmol/L) | 101 (7.03) | 98.6 (7.25) | 103 (6.24) | <0.001 |
| Sodium (mmol/L) | 134 (5.74) | 133 (6.32) | 136 (4.58) | <0.001 |
| Potassium (mmol/L) | 4.66 (0.85) | 4.78 (0.86) | 4.54 (0.82) | 0.039 |
| Glucose (mg/dL) | 209 (109) | 222 (122) | 196 (93.6) | 0.073 |
| INR | 2.08 (1.61) | 2.57 (1.98) | 1.53 (0.71) | <0.001 |
| PT (s) | 22.1 (14.4) | 27.0 (17.5) | 16.6 (6.50) | <0.001 |
| APTT (s) | 60.0 (40.8) | 70.9 (45.4) | 47.7 (30.6) | <0.001 |
| 28day-death | 54 (24.3%) | 38 (34.2%) | 16 (14.4%) | 0.001 |
| ICU-death | 47 (21.2%) | 37 (33.3%) | 10 (9.0%) | <0.001 |
| DIC, events | 29 (13.1%) | 25 (22.5%) | 4 (3.60%) | <0.001 |
| AKI, events | 156 (70.3%) | 87 (78.4%) | 69 (62.2%) | 0.013 |
