# Supplementary material for: Association between antithrombin levels and prognosis in patients with sepsis: a retrospective cohort study based on the MIMIC-IV and MIMIC-III databases
Source: J Intensive Care. 2026 Feb 2;14:24. doi: 10.1186/s40560-026-00862-x (PMC12952102; doi:10.1186/s40560-026-00862-x)
Supplement: Supplementary file 3 [file 40560_2026_862_MOESM3_ESM.docx]

**Table S2.** Characteristics and outcomes of participants stratified by AT-III activity levels in the hypertensive subgroup

| **Characteristic** | **Overall** | **AT-III < 64%** | **AT-III ​≥​ 64%** | **p-value** |
| --- | --- | --- | --- | --- |
|  | ***N=104*** | ***N=52*** | ***N=52*** |  |
| Antithrombin (%) | 62.4 (25.4) | 41.6 (12.0) | 83.1 (16.6) | <0.001 |
| Age (years) | 58.3 (14.8) | 61.8 (14.2) | 54.9 (14.8) | 0.018 |
| Sex: |  |  |  | 1.000 |
| Female | 35 (33.7%) | 17 (32.7%) | 18 (34.6%) |  |
| Male | 69 (66.3%) | 35 (67.3%) | 34 (65.4%) |  |
| Race: |  |  |  | 0.065 |
| White | 55 (52.9%) | 32 (61.5%) | 23 (44.2%) |  |
| Black | 13 (12.5%) | 3 (5.77%) | 10 (19.2%) |  |
| Other | 35 (34.6%) | 16 (32.7%) | 19 (36.5%) |  |
| Weigh (kg) | 93.3 (21.0) | 92.1 (22.0) | 94.5 (20.2) | 0.552 |
| Height (cm) | 172 (8.93) | 172 (9.02) | 173 (8.88) | 0.487 |
| BMI (kg/m^2^) | 31.8 (6.96) | 32.0 (7.19) | 31.6 (6.74) | 0.799 |
| APSIII | 57.4 (22.7) | 66.4 (24.0) | 48.5 (17.3) | <0.001 |
| SAPSII | 40.0 (15.5) | 47.3 (14.9) | 32.8 (12.5) | <0.001 |
| OASIS | 34.2 (8.60) | 36.2 (9.41) | 32.1 (7.23) | 0.014 |
| LODS | 6.52 (3.27) | 7.52 (3.38) | 5.52 (2.86) | 0.002 |
| SOFA | 7.15 (3.79) | 8.71 (3.91) | 5.60 (2.97) | <0.001 |
| CHARLSON | 5.22 (2.47) | 5.58 (2.50) | 4.87 (2.42) | 0.143 |
| Heart Failure | 36 (34.6%) | 20 (38.5%) | 16 (30.8%) | 0.536 |
| Hypertension | 104 (100%) | 52 (100%) | 52 (100%) | . |
| Atrial Fibrillation | 8 (7.69%) | 5 (9.62%) | 3 (5.77%) | 0.715 |
| Diabetes | 45 (43.3%) | 24 (46.2%) | 21 (40.4%) | 0.692 |
| CKD | 33 (31.7%) | 16 (30.8%) | 17 (32.7%) | 1.000 |
| CAD | 33 (31.7%) | 20 (38.5%) | 13 (25.0%) | 0.206 |
| Liver Cirrhosis | 11 (10.6%) | 9 (17.3%) | 2 (3.85%) | 0.056 |
| MAP (/mmHg) | 56.6 (17.3) | 52.5 (18.5) | 60.6 (15.1) | 0.017 |
| SBP (/mmHg) | 85.8 (20.9) | 79.5 (17.2) | 92.0 (22.5) | 0.002 |
| DBP (/mmHg) | 46.7 (12.6) | 43.6 (12.3) | 49.8 (12.4) | 0.013 |
| Temperature (℃) | 110 (22.9) | 114 (23.4) | 106 (21.7) | 0.061 |
| Heart Rate (bpm) | 37.6 (0.86) | 37.5 (0.86) | 37.6 (0.87) | 0.423 |
| Respiratory Rate (/min) | 28.7 (7.26) | 28.7 (6.27) | 28.6 (8.19) | 0.957 |
| Spo2 (%) | 89.9 (10.8) | 87.6 (14.1) | 92.2 (5.16) | 0.030 |
| PH | 7.28 (0.12) | 7.26 (0.14) | 7.31 (0.10) | 0.042 |
| PO2 (/mmHg) | 89.1 (49.1) | 82.6 (43.7) | 97.4 (54.7) | 0.232 |
| PCO2 (/mmHg) | 47.2 (16.1) | 49.7 (18.7) | 44.3 (12.0) | 0.162 |
| Bicarbonate (mmol/L) | 19.7 (5.04) | 19.0 (5.68) | 20.4 (4.21) | 0.154 |
| WBC (×10⁹/L)​​ | 16.0 (7.78) | 18.5 (7.35) | 13.3 (7.40) | 0.001 |
| RBC (×10¹²/L) | 3.75 (0.82) | 3.56 (0.83) | 3.95 (0.77) | 0.017 |
| Platelet (×10⁹/L)​​ | 188 (117) | 157 (124) | 220 (99.9) | 0.005 |
| Hemoglobin (g/dL) | 9.71 (2.50) | 9.04 (2.09) | 10.4 (2.71) | 0.005 |
| Lactate (mmol/L) | 4.45 (4.23) | 6.07 (5.01) | 2.52 (1.65) | <0.001 |
| Creatinine (mg/dL) | 2.41 (2.52) | 2.93 (2.96) | 1.86 (1.84) | 0.029 |
| BUN (mmol/L) | 36.3 (25.5) | 40.4 (26.2) | 32.1 (24.2) | 0.100 |
| ALT (U/L) | 459 (1842) | 630 (2306) | 215 (800) | 0.274 |
| AST (U/L) | 755 (2231) | 1010 (2634) | 393 (1450) | 0.198 |
| TBIL (mg/dL) | 2.76 (5.30) | 3.56 (5.95) | 1.64 (4.05) | 0.095 |
| ALB (g/dL) | 2.82 (0.72) | 2.68 (0.73) | 3.04 (0.67) | 0.068 |
| Calcium (mg/dL) | 7.99 (1.07) | 7.78 (1.14) | 8.23 (0.93) | 0.033 |
| Chloride (mmol/L) | 99.9 (7.23) | 97.8 (7.50) | 102 (6.32) | 0.003 |
| Sodium (mmol/L) | 135 (6.22) | 133 (6.75) | 136 (5.23) | 0.011 |
| Potassium (mmol/L) | 4.74 (0.85) | 4.84 (0.88) | 4.63 (0.83) | 0.216 |
| Glucose (mg/dL) | 231 (129) | 256 (148) | 205 (102) | 0.046 |
| INR | 2.16 (1.76) | 2.63 (2.14) | 1.62 (0.94) | 0.003 |
| PT (s) | 23.3 (18.3) | 28.4 (22.6) | 17.3 (8.09) | 0.002 |
| APTT (s) | 61.1 (44.0) | 70.4 (48.7) | 50.7 (35.8) | 0.027 |
| 28day-death | 27 (26.0%) | 22 (42.3%) | 5 (9.62%) | <0.001 |
| ICU-death | 23 (22.1%) | 20 (38.5%) | 3 (5.77%) | <0.001 |
| DIC, events | 11 (10.6%) | 10 (19.2%) | 1 (1.92%) | 0.011 |
| AKI, events | 74 (71.2%) | 41 (78.8%) | 33 (63.5%) | 0.130 |
